# Educational psychology (2010–2025): living a paradigm change in the study of self-regulated learning behavior

**DOI:** 10.3389/fpsyg.2026.1757975

**Published:** 2026-04-16

**Authors:** Jesús de la Fuente, Douglas F. Kauffman

**Affiliations:** 1 Department of Psychology, School of Education and Psychology, University of Navarra, Pamplona, Spain; 2 Independent Researcher, Boston, MA, United States

**Keywords:** Combined Regulatory Behavior Index (CRBI), models of SRL, self-regulated learning, self-regulation, self-regulation/non-regulation/dysregulation

## Abstract

The course of research into self-regulation (SR) and self-regulated learning (SRL) has shifted over the last fifteen years. Works with an exclusively intra-subject focus have more recently given way to works whose perspective is inter-subject, and the combination of personal and contextual regulation has come to be seen as especially important in explaining SR and SRL. Against that background, it seems worthwhile to describe recent significant advances in the area and to identify aspects that have yet to be fully explored and that require immediate and future investigation. The objectives of this report are: (1) to present the evolution of different relevant theoretical models on self-regulation in learning; (2) to identify the principal reasons for the change and shift in research focus; (3) to identify new paradigms put forward by authors and their applicability; and, (4) to enumerate present and future challenges in this research area. The authors thus hope to provide a synthesis of the development of SR and SR-ER theories, with particular attention to indices of combined behavior regulation (ICR) as a strategic tool for the analysis and evaluation of human behaviors.

## 1 Introduction

The celebration of the fifteenth anniversary of the founding of Frontiers in Psychology is an opportunity for us to contribute a history of a research area. We feel that it is of value to provide from the perspective of today a retrospective and prospective assessment of the course of research in *Self-Regulated Learning* (SRL). Against that background, it is of value to take stock of the significant advances that have been seen in this research topic and to identify issues that deserve present and future endeavor. Consequently, the objective of this research report were: (1) To set out the most significant theoretical models of SRL; (2) to show the key factors behind the change of direction for research; (3) to describe new paradigms and their scope of application; and, (4) to enumerate current and future challenges in the research area. The objective of this research report is not to conduct a systematic review based on an analysis of publications on SRL over the last 15 years. Rather, it is to present the reasons and a narrative explaining the evolution from subject-centered models, when studying SRL, toward combined and interactive individual x context (SRL-ERL) models, necessary to analyze the variance explained by both types of factors.

### 1.1 Relevant theoretical models focused on self-regulated learning (SRL)

A brief review of the evidence that has been generated in research over the last fifteen years confirms that this research topic has continued to be generalized over multiple fields within behavioral research. Thus, the consistent relationships between self-regulation and other adaptive and maladaptive behaviors have been demonstrated within a person-centered approach to gain perspective, it is important to review the relevant conceptual models in the field of self-regulated learning (Pachón-Basallo et al., 2025):

### 1.1.1 Kanfer’s self-regulation model

Frederick Kanfer (1925–2002) was an Austrian engineer, biologist, and psychologist who pioneered the study of self-control and self-regulation in the 1970s. He demonstrated that human behavior is significantly influenced by internal stimuli. Kanfer’s model emerged in response to the limitations of behavioral psychology in explaining individual contributions to self-control and has received substantial empirical support. Kanfer (1970) posited that self-regulation and self-control are fundamental skills for belonging to a community. His model aimed to explain the dynamics when individuals assign themselves reinforcement contingencies in reward experiments. Four fundamental aspects of this model are highlighted (Kanfer, 1977; Kanfer and Hagerman, 1985, 1987).

### 1.1.2 Bandura’s self-regulation model

Albert Bandura (1925–2021), a prominent Canadian psychologist, significantly contributed to the social sciences through his pioneering work on observational learning and social-cognitive theory. He developed a detailed self-regulation model that has influenced subsequent theories and clinical applications (Bandura, 1977a; George-Boeree, 2006; Stewart and Krivan, 2021).

Bandura’s model, rooted in his 1960s research with children, posits that humans are self-regulating and self-reflective organisms, not merely reactive to environmental stimuli (Bandura, 1977a; Stewart and Krivan, 2021; Zimmerman and Schunk, 2003). He proposed the reciprocal determinism model, suggesting that behavior both influences and is influenced by the context, with cognitive factors, personal factors, environmental events, and behavior interacting bidirectionally (Bandura, 1986, 1991). Within *Social-Cognitive Theory*, Bandura (1991) argues that the self-regulatory system mediates external influences and provides the basis for intentional behavior (Kauffman et al., 2023). He describes self-regulatory behavior through three subfunctions: self-monitoring, self-evaluation, and self-reaction These subfunctions depend on personal agency mechanisms such as self-efficacy beliefs (Bandura, 1977a, 1977b, 1986, 1991, 1998, 2005, 2009).

### 1.1.3 Miller and Brown’s self-regulation model

William Miller and Janice Brown, clinical psychologists specializing in addiction behaviors, have been working on self-regulation processes since the early 1990s. They reviewed historical philosophical and social science perspectives, focusing on contributions by Kanfer (1970, 1977), Bandura (1982, 1989), and Deci and Ryan (1987), among others. They aimed to create an integrative self-regulation model incorporating cognitive and volitional factors.

Miller and Brown emphasized that volitional control explains much of the variance in human behavior not accounted for in many psychological studies. They argued that habitual self-regulatory behaviors in the face of temptation enhance volitional control in various situations (Miller and Atencio, 2008; Miller and Brown, 1991). In prevention contexts, they proposed that self-regulation influences behavior by both suppressing risky behaviors and increasing adaptive behaviors (Miller and Brown, 1991).

### 1.1.4 Deci and Ryan’s model: self-determination theory (SDT)

Self-Determination Theory (SDT), developed by psychologists Deci and Ryan at the University of Rochester, is a macro-theory of human motivation and personality with significant implications for understanding human behavior. SDT posits the existence of universal basic psychological needs and social context conditions that contribute to satisfying them, which are central to explaining psychological health, well-being, and human flourishing across various domains.

Key contributions of SDT regard needs and conditions favoring self-determined behavior. These have been relevant for research in education, work, parenting, healthcare, sports, neuropsychology, etc., as they provide theoretical and practical tools to promote self-regulation through self-determined motivational styles (Deci and Ryan, 1987, 2000, 2008, 2014; Ryan and Deci, 2020, 2017; Ryan and Deci, 2020).

### 1.1.5 Zimmerman’s self-regulated learning model (SRL)

Since the late 20th century, the study of self-regulated learning (SRL) has gained significant traction. This research aims to identify the distinctive characteristics that enable individuals to acquire knowledge and skills by integrating affective-motivational and cognitive components (Zimmerman, 1986, 1989a, 1989b, 1990, 1998, 2000, 2001, 2013; Zimmerman and Martinez-Pons, 1986, 1988; Zimmerman and Schunk, 1989, 2003; Zimmerman et al., 2005). Researchers identified the structures underlying students’ abilities to become self-regulated learners and recognized the importance of cognitive, metacognitive, affective, and motivational components as subsystems supporting a volitional “self.” SRL posits that (Zimmerman and Labuhn, 2012):

- Students are creative agents with the power of choice (will),
- Cognitive and metacognitive information processing strategies can be used to achieve personal development goals (skill).

### 1.1.6 Pintrich’s model of self-regulated learning

Paul Pintrich (1954–2003) was a renowned psychologist, professor, and researcher at the University of Michigan. Among his achievements, he served as president of the American Psychological Association (APA) and was a prominent member of the International Association of Applied Psychology (IAAP). His research focused on motivation, epistemological thinking, and self-regulated learning (Limón, 2004; Pintrich, 1989, 1995, 1999, 2000, 2004; Pintrich and Blumenfeld, 1985; Pintrich and De Groot, 1990; Pintrich and Garcia, 1991; Pintrich and Schunk, 1996; Pintrich et al., 1993). Additionally, he collaborated with Bill McKeachie at the National Science Foundation to implement a “learning to learn” program aimed at teaching university students to learn strategies and improve their motivation, leading to his work at the National Center for Research to Improve Postsecondary Teaching and Learning.

### 1.1.7 de la Fuente’s self vs. externally regulated behavior model

Jesús de la Fuente, Professor of Developmental and Educational Psychology (Universities of Almería and Navarra, Spain) has developed an integrative model of self-regulation over three decades, validated in both educational and clinical contexts. This model SRL-ERL (de la Fuente, 2017, 2021; de la Fuente and Kauffman, 2025a), grounded in the contributions of Bandura, Brown, Pintrich, and Zimmerman, is based on Biggs’ 3P Model of Teaching and Learning, for examining the relationships among self-regulation, motivation, and learning strategies. The model distinguishes three levels of regulation (self-regulation, non-regulation, and dysregulation) in both the individual and the context. It considers cyclical phases (preparation, execution, and self-reflection) and analyzes how the combination of personal and contextual regulation levels determines the resulting regulatory tendency (see Table 1).

**Table 1:** Comparative table of the analyzed models (Pachón-Basallo et al., 2025; p. 92).

Features	Functions
Stages/subfunctions/phases	1. Preparation (before) 2. Execution (during) 3. Self-reflection (after)
Relevant variables	- Regulatory taxonomy (self-regulation, non-regulation, dysregulation) - External-regulatory taxonomy - Combination of personal and contextual regulatory characteristics
Contextual approach	- Context plays determining role in regulation - Regulatory characteristics affect subject’s tendencies - Global perception of contexts influences regulation
Key concepts	- Self- vs. external-regulation - Taxonomy of self vs. external-regulation - Combinatorial analysis of self vs. external-regulation: Regulation/de-regulation/dys-regulation

Reproduced with permission.

However, despite the profusion of works following this approach, limitations remain. On the one hand, the lack of clarity as to the level of research at which self-regulation is being used in many studies. On the other hand, excessive focus on intra-subject factors to the detriment of contextual factors and the effects of the subject and their context in combination. Such concerns provided impetus for our research.

## 2 The keys to the development of relevant concepts

### 2.1 Levels of psychological analysis: micro, molecular and molar analysis of self-regulation (SR)

Some works have shown the importance of delimiting the type of analysis in which the concept of self-regulation is used (de la Fuente et al., 2019). Study objectives, the type of analysis performed, and the conclusions reached are mediated by the domain and field of knowledge in which a study is performed. Thus, constructs such as executive functions, self-regulation and self-control have been used in some instances without clear demarcation of their differences and similarities. Simply put, in this domain all those constructs are prevalent. For example, the concept of executive functions is a construct from neuropsychology (behavior microanalysis), whereas self-regulation and self-control are part of clinical psychology and medical practice (molecular behavior analysis).

1. *Microanalysis - neuropsychology*. At this level, executive functions is the variable most deployed in the analysis of behavior self-regulation. Much research at this level has focused on self-regulation and confirmed its importance (Ji et al., 2021; Heinze et al., 2025).
2. *Molecular analysis.* The preferred approach at the molecular level is clinical and health or subject-focused, approaching self-regulation as a personality trait in medical contexts. There is copious evidence in this connection (Cohen, 2024; Daniel et al., 2024; Lambe et al., 2024; López-Martínez et al., 2025).
3. *Molar or contextual and combined analysis:* at this level of analysis, self-regulation is seen as a contextual, interactive variable. Initially, published works sought to analyze the role of self-regulation in the course of learning (Himmelberger et al., 2024; Lee et al., 2025). That approach was generalized to the study of the family environment, showing the relevance of those environments to the self-regulation deployed by individuals (Brown et al., 2017; Orbán et al., 2025; Keyvan et al., 2025). The family environment was shown to be of especial importance in relation to the emotional self-regulation of offspring (Lavi et al., 2021) and their learning strategies (Sasaki and Takeuchi, 2025). See Table 2.

**Table 2:** Levels of research and working formats in the “building” of psychological research. (de la Fuente et al., 2019, p. 3).

Metaphor	Level	Format	Type of variables	Techniques	Models	Implications
3. Journey	Molar (person in context)	Interactive and contextual/applied	Subject x context self-regulation externally-regulation	Observation, self-reports other-reports experiments	SR vs ER	Practices applied to molar and contextualized processes
2. Car	Molecular (person)	Discrete/conceptual personalistic	Subject (Context) meta-attention meta-motivation self-regulation	Observation self-reports experiments	SR (or SRL)	Practices applied to molecular and clinical cases molar processes
1. Engine	Micro-analysis (brain)	Basic/neurological	Brain, microprocesses medication	Tomography, scanner tests clinical cases	EF	Practices applied to basic and molecular processes

Reproduced with permission.

EF = executive functions; SR = self-regulation; SRL = self-regulated learning; SRL vs ERL = self-regulated vs externally regulated learning.

However, despite the copious evidence that now exists, there is a significant limitation in that such works have not used a clear definition of self-regulation as the combined effect of personal and contextual self-regulation. The outcome of that approach was the mission to explain behavior using those constructs alone, albeit some of them did not always explain an acceptable degree of variance. There was thus an evident need to further develop the conceptual underpinnings of the models used.

### 2.2 A molar and combined vision of self-regulated behavior

The consequence of what is laid out above was the emergence of a progressively growing interest in the effect of the *combination* of the person and their context in the study of self-regulated behavior. Thus, there began to appear works that sought to clarify and quantify the weight of context in relation to behavior regulation by individuals.

Researchers began to advance progressively in the exploration of this combined effect of factors of person and context. In relation, for example, to emotions associated with stressful events of everyday life (Hossain et al., 2025), the examination of the effects of contextual factors of teaching on self-regulated learning and emotion and the effect of context and person in combination on regulation of physical activity behaviors (Griffith et al., 2023) and academic stress (de la Fuente et al., 2020).

## 3 The dimension of paradigm change

The realization that there was no integrated model that categorized the different levels of regulatory behaviors gave rise to the development of a systematic model to order those levels hierarchically.

### 3.1 The behavioral typologies continuum: regulation/non-regulation/dys-regulation

The concepts of Self-regulation/non-regulation/dys-regulation (SR-NR-DR) of both the individual and their context were defined in preliminary works. Those initial distinctions were fundamental, because they gave a framework in which to situate prior works concerning the relationships of each level of regulation to other adaptive and maladaptive behaviors (Martínez-Vicente et al., 2025). That differentiation was key to the subsequent development of theoretical models, providing a coherent conceptual framework within which to study possible explanatory mechanisms intrinsic to the individual and their contexts that motivate shifts from one level of behavior to another.

Once it had been satisfactorily established that there are different factors at different levels (of person and context) that combine and interact to explain the variability of behavior regulation and its effects, researchers began to create integrated models to determine such factors in a structured and hierarchical manner. New models were developed that sought to integrate the variability of those behavioral levels of person and context.

### 3.2 The effects of paradigm change: emerging theory

New theoretical model sprang from the reconsideration of earlier theories, that were some what similar but based on a different underlying conceptualization (Pachón-Basallo et al., 2025).

#### 3.2.1 Self-regulated vs. externally regulated learning, SRL-ERL (2017)

**Figure 1:**
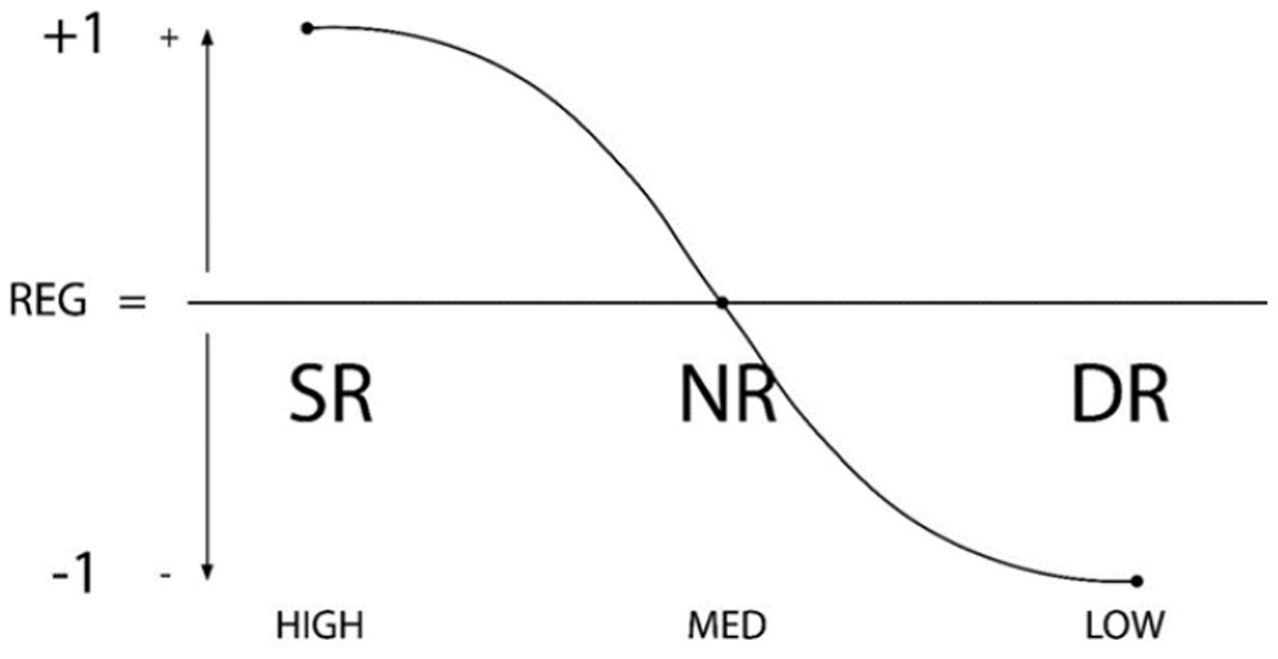
Structural and functional components of SRL graphic representation of regulation types: SR (self-regulation), NR (non-regulation or de-regulation), and DR (dys-regulation). The X axis represents the degree of regulation (high–medium–low), while the Y axis shows directionality (+1, 0, −1) (de la Fuente, 2021; de la Fuente et al., 2022a). Reproduced with permission.

**Figure 2:**
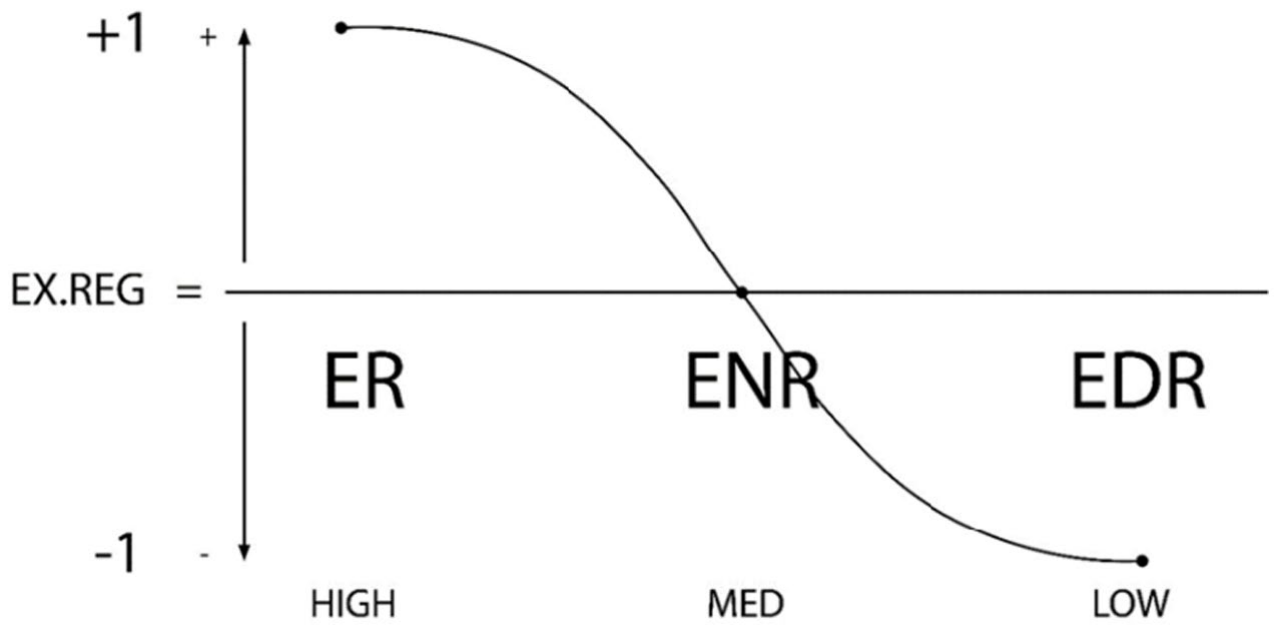
Structural and functional components of ERL. Graphic representation of external regulation types: ER (external regulation), ENR (external non-regulation or deregulation), and EDR (external dys-regulation). The X axis represents the degree of external regulation (high–medium–low), while the Y axis shows the directionality of the external regulation (+1, 0, −1) (de la Fuente, 2021; de la Fuente et al., 2022a). Reproduced with permission.

The first theoretical development was the *Theory of Self- vs Externally- Regulated Learning*™, in educational psychology area (de la Fuente, 2017) as a development of the classic model of SRL (Zimmerman, 2001; Zimmerman and Labuhn, 2012). In parallel, over those early years, research progressed to address the effect of each level of the regulatory continuum of the individual and their environment in relation to other behaviors of academic learning, motivational-affective behaviors, academic achievement and stress and student psychological wellbeing on variables intrinsic to the competency of learning to learn and the impact of ICT use (de la Fuente et al., 2024). Consistent results were found for each level of internal (SR-NR-DR) and external (ER-ENR-EDR) regulation. See Figures 1, 2.

**Figure 3:**
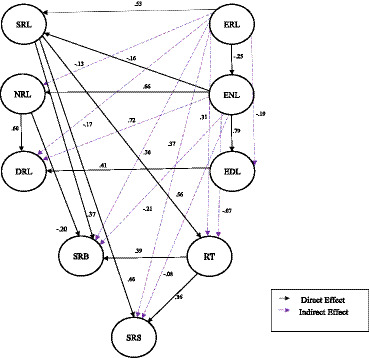
Predictive structural equation model: direct and mediational effects. SRL, self-regulated learning; NRL, non-regulated learning; DRL, dysregulated learning; ERL, externally regulated learning; ENL, externally non-regulated learning; EDL, externally dysregulated learning; SRB, self-regulated behavior; RT, regulatory teaching; SRS, self-regulated study (Pachón-Basallo et al., 2022a, p. 11). Reproduced with permission.

Those results provided evidence to support a general model that applies to motivational-affective behaviors at the molar level, taking a broad view of variables (de la Fuente, 2021). The empirical models explained levels of wellbeing and academic stress and their effect on other personal factors involved in learning (de la Fuente et al., 2021, 2022; Pachón-Basallo et al., 2022a,b). See Figure 3.

#### 3.2.2 The theory of self- vs. externally regulated behavior, SR-ER (2022)

**Figure 4:**
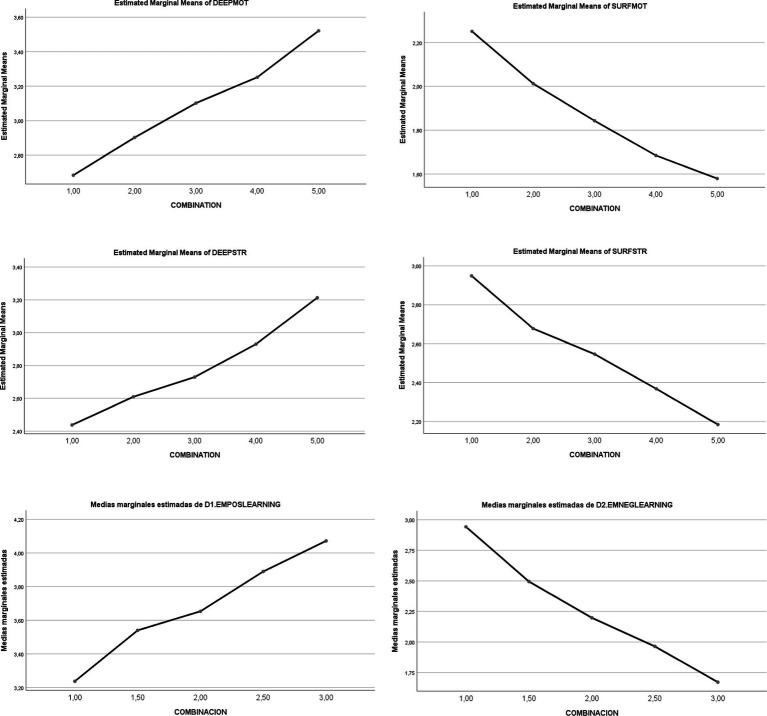
Effects of the combination of SR and ER on different dependent variables. From de la Fuente et al. (2017, p. 240). Reproduced with permission.

Subsequently, that general theoretical model was developed and applied in different areas of psychology, namely education, health and clinical practice, and organizational psychology (de la Fuente et al., 2022a; de la Fuente and Kauffman, 2025a). That entailed the development of a route map for research in those three fields and the creation of the instruments required for the evaluation of the model as applied to them (de la Fuente et al., 2020, 2021, 2022). From that point on, evidence was gathered as to the applicability of the model in other fields, such as educational behavior and health (de la Fuente et al., 2017, 2022; Pachón-Basallo et al., 2022a). See Figure 4.

### 3.3 Seeking a general conceptual framework: the conceptual utility model of management of stress and psychological well-being®

The importance of the paradigm of the combination of person and context and associated theories having been confirmed, there arose the need to have a broader conceptual framework in order to analyze personal and contextual factors that might predict academic stress and psychological wellbeing. This was the genesis of the *conceptual utility model* for the research of academic stress and psychological wellbeing. The conceptual model (de la Fuente and Martínez-Vicente, 2024, 2025; de la Fuente et al., 2022b) is based on a number of principles:

1. The importance of considering different types of variable (presage-process-product), following the 3P model developed by Biggs (2001), in order to analyze the directionality and direct and indirect effects of the variables that explain and predict levels of academic stress and wellbeing.
2. The importance of different factors in combination to prediction of the effect of each type of predicted variable:

1. In terms of levels of presage variables, the model includes Self- vs. External Regulation, in other words personal and contextual regulatory factors in combination (see ICSER).
2. In terms of process variables, the model includes Combined Regulation in the Teaching-Learning Process. It is essential to note as process variables competency for learning and competency for management of stress and wellbeing. At this level of analysis, the model includes variables that are in themselves either protective or risk - in function of their level - relative to the prediction of product variables (see ICBRTL).
3. At the level of product variables, health factors can be considered in weighted combination as regards physical and psychological academic health (see ICAH).

In fact, the selection of some of the most important variables from the conceptual utility model gave rise to the *Index of Experience of Academic Wellbeing* (IEAW) (de la Fuente et al., 2025). And gave rise the AWAB Battery derived from the Index, by way of knowledge transfer of the utility model (de la Fuente et al., 2025a).

## 4 Behavioral combinatory regulatory analysis: a focus on *individual x context* factors

In a subsequent phase, having confirmed the predictive relationships of levels of personal and contextual regulation both individually and in combination, thinking turned to the possibility of studying those factors using *Index of Combined Regulation* (ICR). ICRs have allowed the variability of protective and risk behaviors for learning and mental health/ill health to be ranked and better explained (de la Fuente and Kauffman, 2025b). ICRs are a new strategy for the analysis of personal and contextual factors of self-regulation behavior in combination. The growing number of ICRs and their effects underline the utility of the approach.

### 4.1 Determination of regulatory status via ICRs

Following an initial phase of analysis and categorization of levels of regulation (R-NR-DR), in both the individual (SR-NR-DR) and in their context (ER-ENR-EDR), a heuristic was developed to calculate scores for the different possible combined levels. That score was termed the regulatory status. The different possible combinations were conceptualized and values assigned for their risk vs. protective effect in relation to psychological wellbeing. See Tables 3, 4.

**Table 3:** Levels of regulatory status and of protection and risk.

Regulatory status or level of regulation		Level of protection	Level of risk
High-high	High regulation	High	Low risk
Moderate-high	M-H regulation	Moderate-high	Moderate-low
High-moderate	M-H regulation	Moderate-high	Moderate-low
Moderate	Non-regulation	Moderate	Moderate
Moderate-low	M-L dysregulation	Moderate-low	Moderate-low
Low-moderate	M-l dysregulation	Moderate-low	Moderate-low
Low-low	High dysregulation	low	High

Retrieved from de la Fuente et al. (2019, p. 3).

**Table 4:** Combinations of parameter levels under SRL vs. ERL theory (de la Fuente, 2017; de la Fuente et al., 2019).

Combination of levels		Avg.	Rank	Regulation tendency	Stress protection	Stress risk
SR level (range)	ER level (range)					
3 (3.85–5.00)H	3 (2.84–5.00) H	3.0	5	High-high: high-regulation	High protection	Low risk
2 (3.10–3.84)M	3 (2.84–5.00) H	2.5	4	Medium-high: regulation	M-H protection	M-L risk
3 (3.85–5.00)H	2 (2.35–2.83) M	2.5	4	High-medium: regulation	M-H protection	M-L risk
2 (3–10 - 3.84)M	2 (2.35–2.83) M	2.0	3	Medium: non-regulation	Medium protection	M risk
2 (3.10–3.84)M	1 (1.00–2.34) L	1.5	2	Medium-low: dysregulation	M-L protection	M-H risk
1 (1.00–3.09) L	2 (2.35–2.83) M	1.5	2	Low-medium: dysregulation	M-L protection	M-H risk
1 (1.00–3.09) L	1 (1.00–2.34) L	1.0	1	Low-low: high dysregulation	Low protection	High risk

L, low; M, medium; H, high.

### 4.2 The significance and effect of the different ICRs

#### 4.2.1 General ICR: index of combined internal-external regulation (ICRIE®)

##### 4.2.1.1 Significance

The *Index of Combined Internal-External Regulation,* ICRIE® (de la Fuente and Kauffman, 2025b) is the ICR that allows the determination of the regulatory status of an individual in a specific context: educational, clinical, health or organizational. Each context is evaluated using a specific scale (de la Fuente, 2022; de la Fuente et al., 2022a). That allows calculation of the combined regulation and regulatory status of the individual in each context. The analysis of regulation as a combined process quantifies the different combinations of personal and contextual regulation (See Table 3).

1. *High-high combined regulation*, a high level of both personal and contextual regulation (*high-high combination*). This combination entails that in consequence of both their personal self-regulation and their context the individual has a high level of protection and low level of risk: a high level of competency in managing stress and adversity (high level of adaptive concepts and principles, high level of sustained regulation, high coping strategies, positive emotionality, resilience, personal strengths, engagement, etc.) coupled with low personal and contextual dysregulation, low emotion-based coping strategies, etc. Taken together, those things in turn tend to confer on the individual a high level of psychological wellbeing and flourishing and a low level of stress and negative emotions.
2. *Moderate-high combined regulation*, a moderate-high or high-moderate level of personal and contextual regulation (*high-moderate and moderate-high combinations*). This combination entails that in consequence of both their personal self-regulation and their context the individual has a moderate-high level of protection and low level of risk: a high level of competency in managing stress and adversity (moderate-high level of adaptive concepts and principles, high level of sustained regulation, high coping strategies, positive emotionality, resilience, personal strengths, engagement, etc.) coupled with moderate-low personal and contextual dysregulation, moderate-low emotion-based coping strategies, etc. Taken together, those things in turn tend to confer on the individual a moderate-high level of psychological wellbeing and flourishing and a moderate-low level of stress and negative emotions.
3. *Moderate combined regulation*, a moderate level of both personal and contextual regulation (*moderate–moderate combinations*). This combination entails that in consequence of both their personal self-regulation and their context the individual has a moderate level of protection and low level of risk: a moderate level of competency in managing stress and adversity (moderate level of adaptive concepts and principles, moderate level of sustained regulation, moderate coping strategies, positive emotionality, resilience, personal strengths, engagement, etc.) coupled with moderate personal and contextual dysregulation, moderate emotion-based coping strategies, etc. Taken together, those things in turn tend to confer on the individual a moderate level of psychological wellbeing and flourishing and a moderate level of stress and negative emotions.
4. *Moderate-low combined regulation*, a moderate-low and low moderate level of both personal and contextual regulation (*moderate-low and low-moderate combinations*). This combination entails that in consequence of both their personal self-regulation and their context the individual has a moderate-low level of protection and moderate-low level of risk: a moderate-low level of competency in managing stress and adversity (moderate-low level of adaptive concepts and principles, moderate-low level of sustained regulation, moderate-low coping strategies, positive emotionality, resilience, personal strengths, engagement, etc.) coupled with moderate-high personal and contextual dysregulation, moderate-high emotion-based coping strategies, etc. Taken together, those things in turn tend to confer on the individual a moderate-low level of psychological wellbeing and flourishing and a moderate-high level of stress and negative emotions.
5. *Low combined regulation*, the worst combination of personal and contextual regulation *(low-low combination*). This combination entails that in consequence of both their personality and their context the individual has a very low level of protection and very high level of risk: a low level of competency in managing stress and adversity (low level of adaptive concepts and principles, low level of sustained regulation, low coping strategies, positive emotionality, resilience, personal strengths, engagement, etc.) coupled with high personal and contextual dysregulation, high emotion-based coping strategies, etc. Taken together, those things in turn tend to confer on the individual a low level of psychological wellbeing and flourishing and a high level of stress and negative emotions. See Table 3.

##### 4.2.1.2 Effects found

Some recent studies by our research team have gathered evidence as to the effects of the ICBR on regulatory fatigue and adaptability. That has been investigated with two versions of the ICBR: the classical and a revised version. The level of ICBR has been shown to have an effect on regulatory fatigue, as behavioral mechanism that hinders adaptability. It has been shown that as an individual’s combined regulatory status worsens, their regulatory fatigue and adaptability also worsen, and vice versa (de la Fuente et al., 2025).

#### 4.2.2 The index of combined behavioral regulation in teaching-learning (ICBRTL)

##### 4.2.2.1 Significance

The combinatorial analysis of regulation in TL allows quantification of the different combinations of learning and teaching (See Table 3):

1. *Combined high T-L regulation*, *the combination of a high level of regulation in the teaching and learning processes (High-high T-L combination).* This combination entails that, due both to the student (high self-regulated learning|) and the nature of their context (highly pro-regulatory teaching) the student has a high level of protection and low level of risk: a high level of competency in managing stress and the adversity that arises in academic settings (high level of adaptive concepts and principles, high level of sustained regulation, high coping strategies, positive emotionality, resilience, personal strengths, engagement, etc.) coupled with low personal and contextual dysregulation, low emotion-based coping strategies, etc. Taken together, those things in turn tend to confer on the student a high level of psychological wellbeing and flourishing and a low level of stress and negative emotions.
2. *Combined moderate high T-L regulation, the combination of a high-moderate or moderate-high levels of regulation in the teaching and learning processes (High-moderate and moderate-high T-L combinations).* This combination entails that, due both to the student (learning|) and the nature of their context (teaching) the student has a moderate-high level of protection and low level of risk: a moderate-high level of competency in managing stress and the adversity that arises in academic settings (moderate-high level of adaptive concepts and principles, moderate-high level of sustained regulation, moderate-high coping strategies, positive emotionality, resilience, personal strengths, engagement, etc.) coupled with moderate-low personal and contextual dysregulation, moderate-low emotion-based coping strategies, etc. Taken together, those things in turn tend to confer on the student a moderate-high level of psychological wellbeing and flourishing and a moderate-low level of stress and negative emotions.
3. *Combined moderate T-L regulation*, *the combination of moderate levels of regulation in both teaching and learning processes (Moderate–moderate T-L combination).* This combination entails that, due both to the student and the nature of their teaching context the student has a moderate level of protection and moderate of risk: a moderate level of competency in managing stress and the adversity that arises in academic settings (moderate level of adaptive concepts and principles, moderate level of sustained regulation, moderate coping strategies, positive emotionality, resilience, personal strengths, engagement, etc.) coupled with moderate personal and contextual dysregulation, moderate emotion-based coping strategies, etc. Taken together, those things in turn tend to confer on the student a moderate level of psychological wellbeing and flourishing and a moderate level of stress and negative emotions.
4. *Combined moderate-low T-L regulation, the combination of moderate-low and low-moderate levels of regulation in the teaching and learning processes* (*moderate-low and low-moderate T-L combinations*). This combination entails that in consequence of both their personality and their context the individual has a moderate-low level of protection and moderate-low level of risk: a moderate-low level of competency in managing stress and adversity (moderate-low level of adaptive concepts and principles, moderate-low level of sustained regulation, moderate-low coping strategies, positive emotionality, resilience, personal strengths, engagement, etc.) coupled with moderate-high personal and contextual dysregulation, moderate-high emotion-based coping strategies, etc. Taken together, those things in turn tend to confer on the individual a moderate-low level of psychological wellbeing and flourishing and a moderate-high level of stress and negative emotions.
5. *Combined low T-L regulation, the worst combination of levels of regulation in the teaching and learning processes* (*Low-low T-L combination*). This combination entails that, due both to the student and the nature of their teaching context the student has a low level of protection and high level of risk: a low level of competency in managing stress and the adversity that arises in academic settings (low level of adaptive concepts and principles, low level of sustained regulation, low coping strategies, positive emotionality, resilience, personal strengths, engagement, etc.) coupled with high personal and contextual dysregulation, high emotion-based coping strategies, etc. Taken together, those things in turn tend to confer on the student a low level of psychological wellbeing and flourishing and a high level of stress and negative emotions.

##### 4.2.2.2 Effects found

In this case, research has also shown a graduated effect on positive and negative emotions and on psychological wellbeing itself in university settings (de la Fuente et al., 2020).

## 5 Future applications of the combinatory *person x context* paradigm (SR-ER)

The combinatory heuristic has broad scope for future application. It could be extrapolated to different uses, depending on the field.

### 5.1 SR-ER in educational psychology (SRL-ERL)

In *Educational Psychology* the paradigm helps us to understand different regulatory statuses of the student in interaction with their context, the teaching-learning process and their mental-physical health. Taken together, they form the total score for the IEBEA (de la Fuente et al., 2025). The indices support the choice of intervention at different levels of combined regulatory status and mental-physical health.

#### 5.1.1 Combination of student plus context

1. *Improved behavior regulation in students*. The heuristic presented here allows determination of the roles of different variables in psychological wellbeing, such as regulatory fatigue (Martínez-Vicente et al., 2025), procrastination (Garzón-Umerenkova et al., 2025), and ADHD in students (de la Fuente and Kauffman, 2025a). To that end, the *Programa Pro & Regula* [*Pro and Regula Program*] (Martínez-Vicente and de la Fuente-Arias, 2004) provides a simple tool to improve self-regulation behavior in its different phases of education based on Zimmerman (2001) model.
2. *Improved regulatory characteristics of the family and social context of students*. Educational activity can be carried out with families to encourage more positive parenting, both through direct interventions with families and indirectly through schools through Programs of Psychological Education (de la Fuente, 2022; de la Fuente et al., 2024), as well as through advice via glossed texts (de la Fuente, 2022).

#### 5.1.2 Combination in the teaching-learning process

1. *Improved learning process.* Intervention at this level specifically requires improved learning strategies and greater self-regulation of learning as well as competency in the management of academic stress (de la Fuente et al., 2022a). Teaching planning, self-observation and self-control together with strategies for self-evaluation in the course of the learning process is an essential improvement tool (Sánchez-Roda et al., 2007).
2. *Improved teaching process*. At the same time, improvements to teaching such to make it more pro-regulatory and so support better learning, is an important and necessary strategy.
3. *Improved physical health*. This type of preventive intervention will help students manage chronic diseases and adopt better health habits: diet, exercise, posture in the classroom, avoidance of unhealthy substances, etc.
4. *Improvement of psychological health*. In this case, the intervention will be aimed at providing students with specific skills in positive health, coping strategies, emotional management, emotional intelligence and psychological well-being.

This broad, combinatory perspective allows the analysis of the predictive, mediating role of different factors in the psychological wellbeing of students. See Table 5 and Supplementary material.

**Table 5:** Conceptual utility model for the management of stress and psychological wellbeing, CMSPW (de la Fuente and Martínez-Vicente, 2024; IPR no 00765-01162097) as applied in educational psychology.

Level	Presage variables	Process variables		Product variables
Personal	Sex/age Personality Self-regulation in educational contexts	Conceptual	Learning focuses and styles	Academic achievement Academic satisfaction Academic stress Flourishing Academic health Psychological wellbeing
		Procedural	Cognitive and metacognitive learning strategies Coping strategies Self-regulation Self-control Engagement Procrastination Emotional dysregulation Motivational habits and strategies Executive functions *Burnout*	
		Attitudinal	Adaptability Resilience Achievement emotions Action-emotion style Reactance Perfectionism Personal strengths Test anxiety Academic confidence Study habits	
Contextual	External regulation in academic contexts Family, social support	Implementation of teaching process Experience of teaching-learning processes Academic stressors in the teaching process		

Taken from R&D Project ref. PID2022-136466NB-I00. https://www.inetas.net/stress/seccion.php?ididioma=2&idseccion=1&idproyecto=10.

## 6 Conclusion, limitations and prospectives

### 6.1 Conclusion

The creation of new theories and predictive-explanatory models is the best psychological antidote to black-box thinking based on unsubstantiated notions of mental health (Castro, 2024). That is, the paradigm shift that the authors have laid out responds to the imperative to develop new theoretical frameworks that are capable of empirical challenge and validation as an integral part of the science of psychology. Moreover, the explanatory impetus that underlies this paradigm shift is exactly to better explain mental health and psychological wellbeing in the different fields of assessment and intervention canvased above. An example is the application of the SR-ER theoretical model to the issue of the assessment and improvement of the emotional wellbeing of influencers, with promising approaches (de la Fuente et al., 2025b).

This new focus of analysis has generated new notions and terminology that future research must validate. Thus, just as it has been possible to provide precise meanings for regulation/non-regulation/dysregulation, it now seems plausible to look to extend the underlying concept of continua to other issues. Thus, for example, we could use the concepts of *love*/*de-love*/*dys-love to* describe different romantic behaviors, from the most adaptive to the most pathological (de la Fuente et al., 2025a, 2025b). It would also be possible to frame an adaptation/non-adaptation/dysadaptation continuum to denote different levels of adaptive behavior (de la Fuente et al., 2025).

### 6.2 Limitations

Several limitations must be acknowledged in this research report. The first concerns the excessive focus on the theoretical and empirical output of this line of research. This is justified by the objective of demonstrating the foundations and innovations within this field. The second limitation is the reliance on university-based samples for the results, which restricts the generalizability of the model to other contexts. The third -and no less important-limitation is the lack of contrast analyses between the presented SRL-ERL model and the theoretical models previously discussed. Some of these studies are already underway, but we cannot present empirical results at this time. We will provide a full account of them in the near future.

### 6.3 Prospectives

There are many challenges to be faced in the future. On one hand, the development of the paradigm opens the door to molar-molecular and microanalytic analyses. On the other hand, specific therapeutic approaches will be developed, both for individuals and for their contexts (de la Fuente et al., 2025b). New mediational and variable modeling studies have taken SR-ER as presage variable, intermediate variables as process or mediating variables and product variables as final dependent variables (de la Fuente et al., 2025b).

We invite interested researchers to consider the theoretical model more widely, to posit hypotheses and to test those hypotheses empirically in whatever their field of study. Only in that way can we determine the limits of these new theoretical structures and how far their results can be empirically generalized to different fields of application globally. We would be delighted to add them to our international network (www.inetas.net).

## Data Availability

Publicly available datasets were analyzed in this study. This data can be found at: www.inetas.net.

## In memoriam

This work is dedicated to our admired Barry J. Zimmerman (1942–2025).
